# The effect of diet on the gastrointestinal microbiome of juvenile rehabilitating green turtles (*Chelonia mydas*)

**DOI:** 10.1371/journal.pone.0227060

**Published:** 2020-01-15

**Authors:** Jennifer C. G. Bloodgood, Sonia M. Hernandez, Anitha Isaiah, Jan S. Suchodolski, Lisa A. Hoopes, Patrick M. Thompson, Thomas B. Waltzek, Terry M. Norton

**Affiliations:** 1 Daniel B. Warnell School of Forestry and Natural Resources, University of Georgia, Athens, Georgia, United States of America; 2 Southeastern Cooperative Wildlife Disease Study, College of Veterinary Medicine, University of Georgia, Athens, Georgia, United States of America; 3 Gastrointestinal Laboratory, College of Veterinary Medicine and Biomedical Science, Texas A&M University, College Station, Texas, United States of America; 4 Georgia Aquarium, Atlanta, Georgia, United States of America; 5 Department of Infectious Diseases and Immunology, College of Veterinary Medicine, University of Florida, Gainesville, Florida, United States of America; 6 Georgia Sea Turtle Center, Jekyll Island Authority, Jekyll Island, Georgia, United States of America; IRIG-CEA Grenoble, FRANCE

## Abstract

Threatened and endangered green turtles (*Chelonia mydas*) are unique because as juveniles they recruit from pelagic to near-shore waters and shift from an omnivorous to primarily herbivorous diet (i.e. seagrass and algae). Nevertheless, when injured and ill animals are admitted to rehabilitation, animal protein (e.g. seafood) is often offered to combat poor appetite and emaciation. We examined how the fecal microbiome of juvenile green turtles changed in response to a dietary shift during rehabilitation. We collected fecal samples from January 2014 –January 2016 from turtles (N = 17) in rehabilitation at the Georgia Sea Turtle Center and used next generation sequencing to analyze bacterial community composition. Samples were collected at admission, mid-rehabilitation, and recovery, which entailed a shift from a mixed seafood–vegetable diet at admission to a primarily herbivorous diet at recovery. The dominant phyla changed over time, from primarily Firmicutes (55.0%) with less Bacteroidetes (11.4%) at admission, to primarily Bacteroidetes (38.4%) and less Firmicutes (31.8%) at recovery. While the microbiome likely shifts with the changing health status of individuals, this consistent inversion of Bacteroidetes and Firmicutes among individuals likely reflects the increased need for protein digestion, for which Bacteroidetes are important. Firmicutes are significant in metabolizing plant polysaccharides; thus, fewer Firmicutes may result in underutilization of wild diet items in released individuals. This study demonstrates the importance of transitioning rehabilitating green turtles to an herbivorous diet as soon as possible to afford them the best probability of survival.

## Introduction

Research in the last decade has firmly established that the gastrointestinal (GI) microbiota is intimately tied to vital aspects of health, including the production of vitamins and essential amino acids and the utilization of fat and glucose [1]. Commensal microbes also play important roles in host immunity and serve as barriers to pathogenic bacterial colonization [2]. In humans, disruption to the GI microbiota is associated with problems such as obesity, inflammatory bowel disease, infectious gastroenteritis and colitis, and diabetes [1, 3]. With improved understanding of this relationship to health and disease, the focus is shifting to the processes that influence microbiome composition. Several links have been established, including host phylogeny, physiology, diet, and antimicrobial exposure [4–6]. The influence of diet, in particular, is gaining attention because of the potential to manipulate it to favor the growth of particular bacteria to improve health [7].

Despite considerable advancements in the study of the GI microbiota and a seemingly exponential increase in the number of studies in various taxa of animals, the reptilian microbiome remains poorly understood [8]. Knowledge about the microbiome of sea turtles, all species of which are listed internationally as either Threatened or Endangered [9], is limited but expanding [10–16]. Understanding endangered sea turtle core microbiota and what influences its composition is likely to influence how we manage conservation efforts. Green turtles (*Chelonia mydas*) are a particularly interesting subject for GI microbiota research because their dietary requirements are unique among sea turtles. Hatchlings and pelagic juveniles are omnivorous, and after recruiting to coastal waters, older juveniles and adults are primarily herbivorous. Indeed, the microbiome of pelagic juveniles is different from that of coastal juveniles in the Northern Gulf of Mexico, suggesting influences from both habitat and diet [14]. In the western Atlantic, this ontogenetic shift occurs when individuals reach 20 to 25 cm carapace length [17], however the timing and extent of this shift varies among other populations of green turtles [18–23].

Understanding the effects of this unique foraging ecology on the GI microbiota of green turtles is important for two reasons: 1) significant associations of the microbial composition with their health might inform specific management actions to protect habitat, and 2) injured and sick sea turtles are frequently rescued and rehabilitated in specialized hospitals where they are fed various diets that may influence their microbiome. Limited research exists on the consequence of diet fed during rehabilitation on the GI microbiome and the impact this may have on recovery, time to release, and long-term survival after release. Studies have shown that the green turtle microbiome differs between wild and stranded individuals, as well as in individuals pre- and post-rehabilitation [11, 12]. Disruptions to the microbiome in turtles post-rehabilitation was attributed in part to diet, as turtles were fed strictly seafood with no variation in diet throughout rehabilitation [12]. In contrast to that study, however, many hospitals initially feed food items high in animal protein (e.g. fish, squid, shrimp) to combat poor appetite and emaciation, but attempt to switch individuals to a vegetable-based diet before release to more closely mimic their wild diet. The effects of this practice on the GI microbiota has not been studied. Understanding how the microbiome responds to these dietary changes will allow managers to best design nutritional protocols that most closely support the microbiome found in healthy, wild green turtles, and potentially speed recovery and release back into natural environments.

The objective of this study was to describe, for the first time, the genotypic bacterial community composition of feces from juvenile green turtles over time with changes in diet during rehabilitation. We expected a strong correlation between changes in GI microbial diversity and composition with changes in diet during the rehabilitation period. Here we present our results and implications for green turtles in rehabilitation facilities that will be released back to the wild, as well as those that are held in more permanent aquaria.

## Materials and methods

### Study site and animals

Fecal samples were collected from January 2014 –January 2016 from juvenile green turtles admitted for rehabilitation at the Georgia Sea Turtle Center (GSTC) on Jekyll Island, Georgia, USA (31.07°N, 81.41°W). Samples were collected at three time points (admission, mid-rehabilitation, and recovery) defined according to the diet consumed. At each time point, physical exams were performed. A subjective body condition score (BCS) on a scale of 1–5 was recorded. Turtle weight and size, measured as straight carapace length (SCL), were also recorded, and body condition index (BCI) was calculated from this information [24]. Antibiotic administration (type, duration) was carefully recorded.

The turtles were maintained in 8 feet (2.43 m) x 8 feet (2.43 m) circular custom-made fiberglass tanks in salt water at a temperature of approximately 75°F (24°C). Turtles were often co-housed, but dividers were in place to prevent feces from moving between individual’s spaces. A sophisticated closed filtration system was utilized for each set of tanks, which consisted of a protein skimmer, biological filter, bead filter and ozone for disinfection. Enrichment was provided throughout the rehabilitation process, including PVC bottom feeders for vegetables fed, shade cloth over portions of the tank for hiding, weighted hide boxes at the bottom of the tank, PVC back scratchers, and occasional lasso feeding to increase movement in the tank.

All capture, handling and sampling procedures were approved by the following: GSTC IACUC approval (#2013–1), Georgia Department of Natural Resources Scientific Collecting Permits (#29-WJH-13-140, #29-WJH-14-201, 29-WJH-15-161, #29-WJH-16-214), and/or Florida Fish and Wildlife Conservation Commission Marine Turtle Permits (#MTP-14-135, #MTP-15-135A, #MTP-16-135A).

### Rehabilitation diets

Food items fed to green turtles at the GSTC included romaine lettuce and leafy lettuce (*Lactuca sativa*), cucumber (*Cucumis sativus*), green bell pepper (*Capsicum annuum*), mackerel (*Scomber scombrus*), herring (*Clupea harengus*), shrimp (*Penaeus* spp.), squid (*Loligo opalescens*), and a custom gelatin-based diet consisting of vegetables, seafood, and vitamins [25]. In addition, multivitamin (Mazuri^®^ Vita-Zu^®^ Sea Turtle Vitamin for Fish-based diets, 500mg, catalog number 1815523–300, Mazuri, Richmond, IN, USA) and calcium supplements (Calcium Carbonate 10 gr, 648mg, catalog number 00536-1024-10, Rugby, Livonia, MI, USA) were offered daily in food items to ensure consumption. For this study, three sampling time points were defined by diet. “Admission” samples were taken as soon as possible after presentation to the hospital, when turtles were offered a mixed seafood and vegetable diet. Over time, individuals were transitioned to a higher percentage of vegetables. “Mid-rehabilitation” samples were collected after individuals consumed 25% vegetables for at least 2 weeks, and “recovery” samples were collected after individuals consumed 50% vegetables for at least 2 weeks. Individual turtles varied in how long they took to transition to the mid-rehabilitation and recovery diets. Detailed records were maintained on the amounts of each diet item offered and consumed at each feeding.

### Sample collection and processing

Fecal samples were collected from the tanks within 4 hours of defecation. Fecal pellets were broken open and a sterile swab was placed in the core of the sample to minimize collecting feces exposed to salt water from the tank. The swab was then placed in 0.5 mL of RNA*later*^®^ (ThermoFisher Scientific, Waltham, MA, USA), refrigerated, and later frozen at -80°C within 24 hours.

To test for the effects of time spent in salt water on the fecal microbiota, a subset of turtles (N = 2) were monitored and feces were collected and swabbed immediately after defecation. Feces were also transferred to a container of salt water from the tank, and subsequent swabs were collected after 1, 5, 15, 30, 60, 120 and 240 min. This time-series analysis indicated that bacterial community composition did not change significantly over time (S1 Fig). Thus, although attempts were made to collect the samples as soon as possible, it was considered satisfactory to collect samples within four hours of defecation.

Samples were randomized prior to extraction using “research randomizer” (www.randomizer.org/). DNA was extracted using a DNeasy Blood and Tissue Kit (Qiagen Ltd., West Sussex, England) following the manufacturer’s instructions. After extraction, fluorometric quantitation was used to confirm presence of DNA (Qubit^™^ 3.0 Fluorometer, ThermoFisher Scientific), and extracts were stored at -80°C until further analysis.

Polymerase chain reaction (PCR) and sequencing steps broadly followed the Illumina 16S protocol (16S Metagenomic Sequencing Library Preparation; URL: http://www.illumina.com/content/dam/illumina-support/documents/documentation/chemistry_documentation/16s/16s-metagenomic-library-prep-guide-15044223-b.pdf). The V3 and V4 regions of the bacterial 16S rRNA gene were amplified by PCR (95°C for 3 min, followed by 30 cycles at 95°C for 30 sec, 55°C for 30 sec, 72°C for 30 sec, and a final extension at 72°C for 5 min) using the following primers: 5'-TCGTCGGCAGCGTCAGATGTGTATAAGAGACAGCCTACGGGNGGCWGCAG-3' and 5'-GTCTCGTGGGCTCGGAGATGTGTATAAGAGACAGGACTACHVGGGTATCTAATCC-3'. Positive (*Salmonella enteritis*) and negative (molecular grade water) controls were included for all PCR reactions. The size of the PCR products was verified by gel electrophoresis. A 50μL index PCR reaction was then completed (95°C for 3 min, followed by 8 cycles at 95°C for 30 sec, 55°C for 30 sec, 72°C for 30 sec, and a final extension at 72°C for 5 min) using the library primers from the Nextera XT DNA Library Preparation Kit (Illumina). The presence and size of the PCR products were again verified by gel electrophoresis, and fluorometric quantitation was used to quantify the concentration of each library (Qubit^™^). Amplicon concentrations were normalized, pooled, and sequenced on an Illumina MiSeq (San Diego, CA, USA).

### Data analysis

Pre-processing of the raw sequence data was performed using the QIIME pre-processing application within Illumina basespace (https://basespace.illumina.com) [26]. Operational Taxonomic Units (OTUs) were assigned based on at least 97% sequence similarity against the Greengenes reference database [27]. For downstream analysis, sequences assigned as Chloroplast, Mitochondria and Unassigned were removed. Sequences were rarefied to an even depth of 9000 sequences per sample to account for unequal sequencing depth across samples. Rarefaction curves showing alpha diversity indices (Chao1, Shannon and Observed OTUs) and beta-diversity analysis using principal coordinate analysis (PCoA) plots and weighted and unweighted UniFrac distance metrics were generated within QIIME 1.9 [28]. QIIME 1.9 has recently been replaced by QIIME 2.0, however the authors do not feel that analysis using QIIME 1.9 reduces the reliability of the conclusions drawn in this study, and allows comparison to other studies using similar versions of the program [12, 13].

Data for summary statistics of bacterial taxa and alpha-diversity measures were tested for normality using the Shapiro-Wilk test (JMP Pro 11, SAS software Inc., Cary, NC, USA). Most datasets did not meet the assumptions of normality, hence Friedman’s test within was used (Prism v .5.0, GraphPad Software Inc., San Diego, CA, USA). Benjamini & Hochberg’s False Discovery Rate was used to adjust the resulting p-values for multiple comparisons, and an adjusted p<0.05 was considered statistically significant. Multiple linear regression was used to determine if capture location, number of days in rehabilitation, SCL, or BCI significantly predicted Shannon diversity index values at admission and recovery. Additionally, a linear discriminant analysis effect size (LEfSe) algorithm (Calypso v8.72, http://cgenome.net/calypso/) was used to elucidate the bacterial taxa with significant differential relative abundances associated with time points [29].

Analysis of Similarity (ANOSIM) was used to analyze significant differences in bacterial communities across samples (PRIMER-E Ltd., Luton, UK). R-values of the ANOSIM test range from -1 to +1 and are an estimate of the effect size. Values closer to zero indicate the samples are similar to each other, and values closer to 1 indicate the samples are dissimilar.

Mann-Whitney tests and ANOSIM were used to analyze the effect of antibiotic usage on alpha and beta diversity. Because most turtles in this study had received antibiotics before or at the time of the mid-rehabilitation and recovery time points, only the admission time point was analyzed for an effect of antibiotics.

### Information on deposited data

The sequences were deposited in the Sequence Read Archive database of the National Center for Biotechnology Information (NCBI; Accession no. SRP080996).

## Results

### Samples and turtles

Fecal samples were collected at all three time points from 17 individual turtles (N = 51). Of these, 9 were admitted from Florida, six from Georgia, and two from Massachusetts. The average SCL of these individuals was 29.9 cm (range 23.1–37.0 cm). The time to collect feces varied among individuals. The admission sample was collected as soon as possible after admission, however, some turtles were anorectic and not producing feces, thus the average time in rehabilitation prior to fecal collection was 10 days (range 0–68 days). For the mid-rehabilitation sample it was 54 days (range 25–114 days), and for the recovery sample it was 109 days (range 49–231 days) (Table 1).

**Table 1:** History and physical exam data for green turtles in rehabilitation at the Georgia Sea Turtle Center at the three timepoints of fecal collection for metagenomic analysis (admission, mid-rehabilitation, and recovery)^a^.

Turtle ID	Time point	Date	Days in Rehabilitation	SCL	BCI	BCS	Capture Location
A	Admission	01/10/14	4	30.2	1.1	2.00	Florida
	Mid-rehab	04/02/14	86	31.5	1.4	3.00	
	Recovery	05/06/14	120	33.1	1.4	3.50	
B	Admission	01/13/14	4	34.5	1.3	2.50	Florida
	Mid-rehab	02/26/14	48	34.4	1.4	3.50	
	Recovery	05/19/14	130	36.2	1.3	3.75	
C	Admission	01/15/14	5	33.8	1.0	1.75	Georgia
	Mid-rehab	02/21/14	42	34.5	1.1	3.00	
	Recovery	03/09/14	58	35.1	1.2	3.75	
D	Admission	02/02/14	6	37.0	1.2	2.00	Georgia
	Mid-rehab	02/23/14	27	37.4	1.3	3.50	
	Recovery	03/31/14	63	38.1	1.3	3.50	
E	Admission	01/31/14	1	27.0	1.1	1.50	Florida
	Mid-rehab	02/28/14	29	27.0	1.0	2.00	
	Recovery	03/21/14	50	28.3	1.1	2.75	
F	Admission	02/03/14	3	32.5	1.2	2.50	Georgia
	Mid-rehab	02/26/14	26	32.7	1.0	2.50	
	Recovery	03/21/14	49	34.0	1.2	3.00	
G	Admission	02/08/14	1	33.2	1.1	2.25	Georgia
	Mid-rehab	03/04/14	25	33.2	1.1	2.25	
	Recovery	05/04/14	86	34.8	1.3	3.75	
H	Admission	04/03/14	0	NA	NA	1.75	Florida
	Mid-rehab	05/18/14	45	NA	NA	2.50	
	Recovery	08/21/14	140	28.1	1.4	3.50	
I	Admission	10/06/14	1	26.5	1.2	2.00	Florida
	Mid-rehab	11/10/14	36	NA	NA	3.50	
	Recovery	05/12/15	219	31.0	1.2	3.75	
J	Admission	10/08/14	0	25.6	1.2	3.00	Florida
	Mid-rehab	12/29/14	82	NA	NA	4.00	
	Recovery	05/27/15	231	29.3	1.4	4.00	
K	Admission	11/28/14	8	27.2	1.3	3.00	Georgia
	Mid-rehab	03/14/15	114	NA	NA	3.50	
	Recovery	05/22/15	183	30.5	1.3	3.75	
L	Admission	05/28/15	68	30.4	1.1	2.00	Florida
	Mid-rehab	06/15/15	86	NA	NA	2.50	
	Recovery	07/21/15	122	33.4	1.3	3.50	
M	Admission	05/03/15	20	30.2	1.3	2.50	Florida
	Mid-rehab	05/12/15	29	NA	NA	2.50	
	Recovery	06/04/15	52	30.9	1.2	3.00	
N	Admission	01/16/16	35	23.1	1.3	3.00	Massachusetts
	Mid-rehab	02/20/16	70	NA	NA	3.00	
	Recovery	03/10/16	89	27.0	1.4	3.50	
O	Admission	12/12/15	1	23.6	1.2	2.50	Massachusetts
	Mid-rehab	02/08/16	59	NA	NA	NA	
	Recovery	03/10/16	90	25.5	1.3	3.00	
P	Admission	02/03/16	13	32.8	1.3	2.25	Georgia
	Mid-rehab	03/01/16	40	NA	NA	2.50	
	Recovery	04/09/16	79	34.3	1.3	3.50	
Q	Admission	01/31/16	6	30.5	1.2	2.25	Florida
	Mid-rehab	04/16/16	82	NA	NA	3.50	
	Recovery	04/28/16	94	35.2	1.2	3.50	

^a^SCL: straight carapace length; BCI: body condition index; BCS: body condition score; NA: not assessed

### Illumina sequencing output

The MiSeq run generated a total of 24,521,956 quality reads. After pre-processing in QIIME, 3,497 unique OTUs were identified across all 51 samples. The bacterial communities largely consisted of Firmicutes and Bacteroidetes, followed by Proteobacteria.

### Bacterial diversity across time points

**Fig 1:**
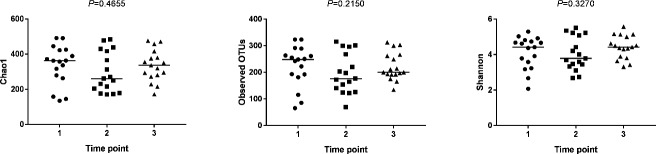
Juvenile green turtle fecal bacterial alpha diversity across time points in rehabilitation. Alpha diversity indices (Chao1, Observed OTUs, and Shannon Diversity Index) showed no significant differences in juvenile green turtle (*Chelonia mydas*) fecal bacterial diversity across time points in rehabilitation (1 = admission, 2 = mid-rehabilitation, 3 = recovery).

**Fig 2:**
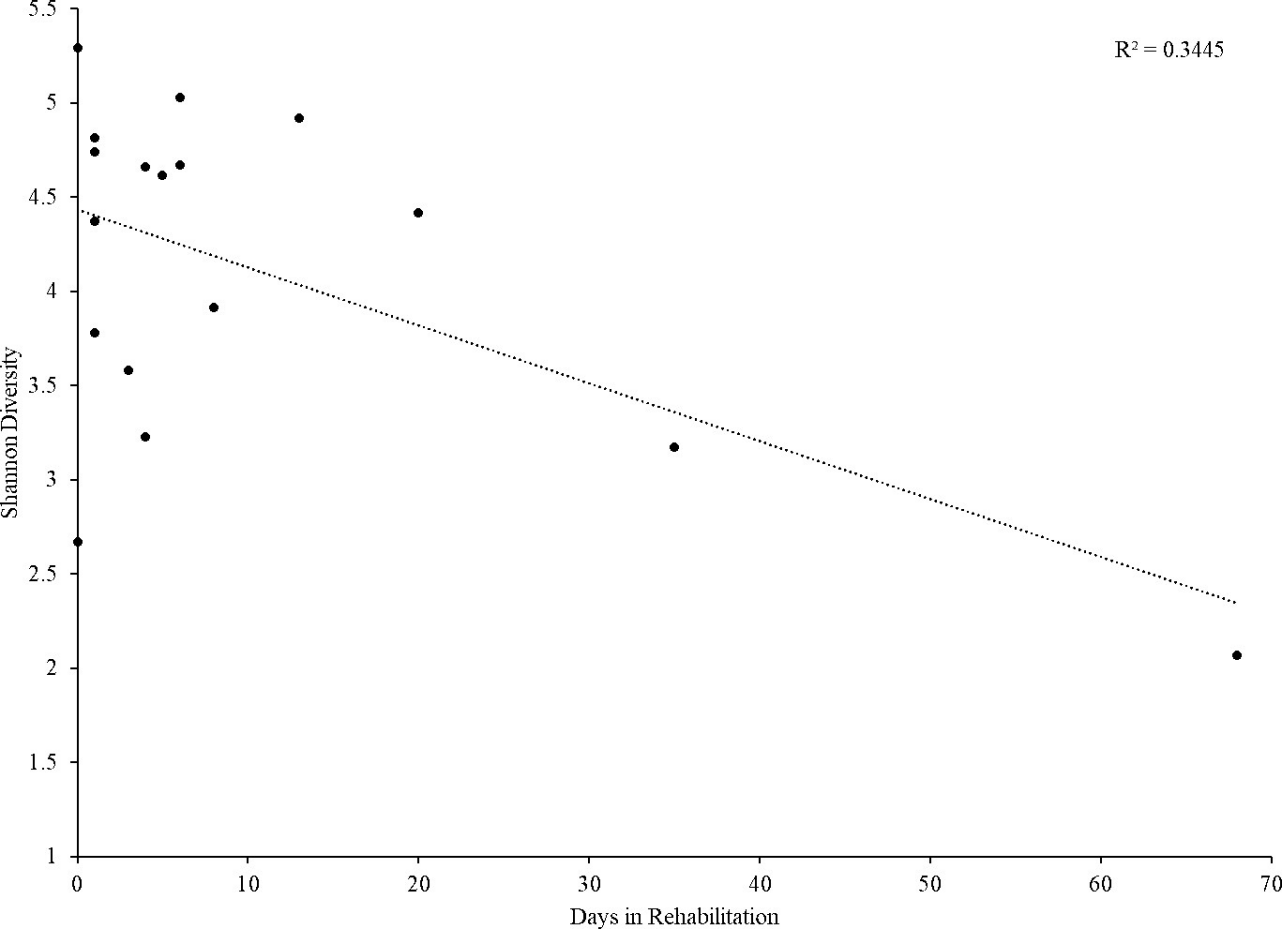
Shannon diversity values of admission samples by number of days in rehabilitation. Shannon diversity decreased significantly with increasing number of days in rehabilitation at the admission time point (p = 0.013).

Alpha diversity (Chao 1, observed OTUs, and Shannon diversity index) was not significantly different across time points (Fig 1). Multiple regression showed that capture location, number of days in rehabilitation, SCL (an approximation of age in chelonians), and BCI did not significantly predict Shannon diversity levels of bacteria at admission and recovery (p = 0.124 and p = 0.482, respectively). When simple linear regressions were utilized, however, there was a significant decrease in bacterial diversity with increasing days in rehabilitation, but only at the admission time point (Fig 2; p = 0.013).

**Fig 3:**
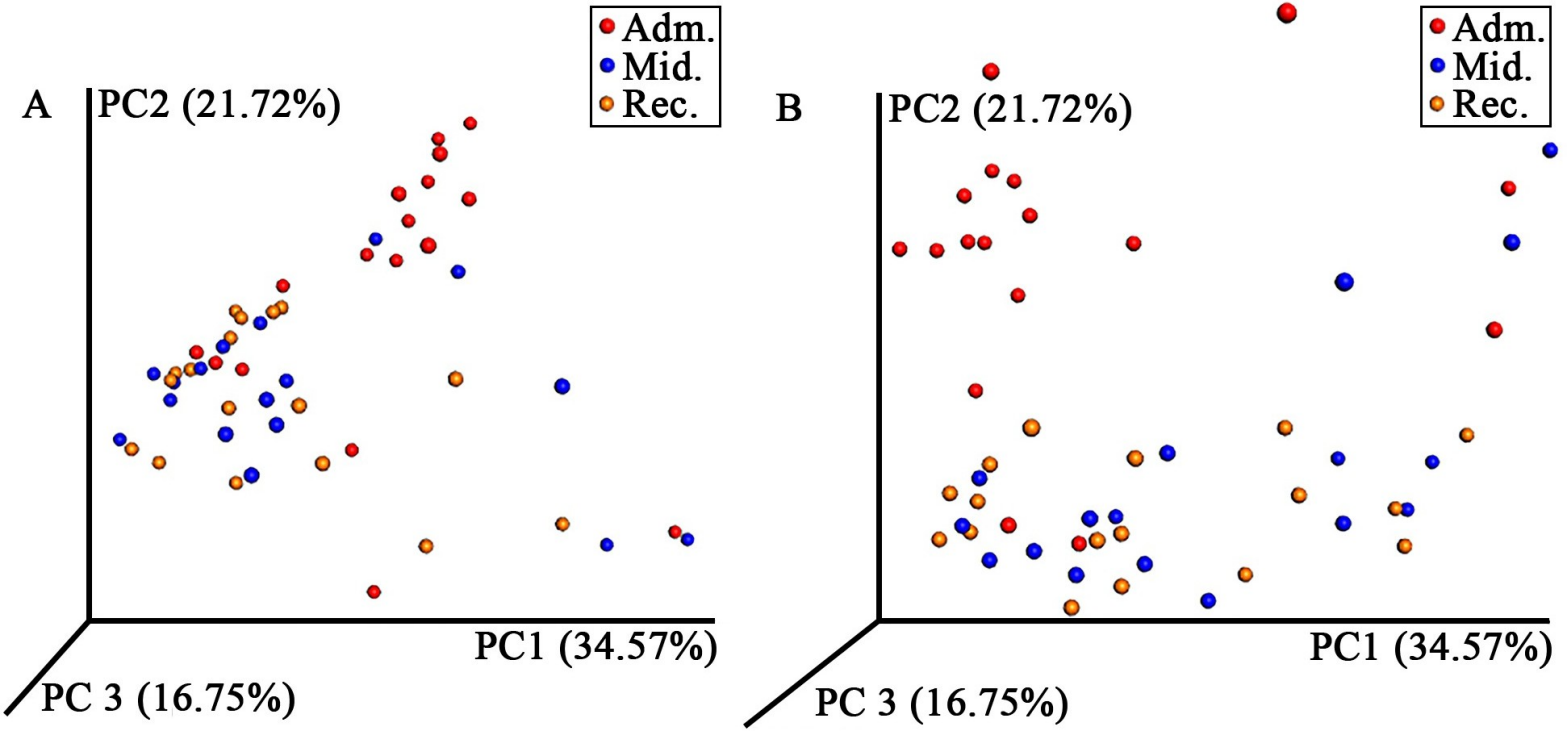
Juvenile green turtle fecal bacterial community composition across three time points in rehabilitation. Principle coordinate analysis of weighted (A) and unweighted (B) UniFrac distances of 16S rRNA genes found in feces from juvenile green turtles (*Chelonia mydas*) at various time points during rehabilitation (red = admission, blue = mid-rehabilitation, and yellow = recovery). Each circle represents an individual fecal sample. Composition at admission was significantly different from mid-rehabilitation (A: R = 0.121, p = 0.006; B: R = 0.258, p = 0.001). Composition at admission was also significantly different from recovery (A: R = 0.222, p = 0.001; B: R = 0.323, p = 0.001). Bacterial composition at mid-rehabilitation and recovery were not significantly different (A: R = -0.022, p = 0.713; B: R = -0.017, p = 0.642).

There were significant differences in bacterial community composition among some time points (Fig 3; p = 0.001). Composition at admission was significantly different from mid-rehabilitation and recovery, while bacterial composition at mid-rehabilitation and recovery were not significantly different.

### Taxonomic composition across time points

**Fig 4:**
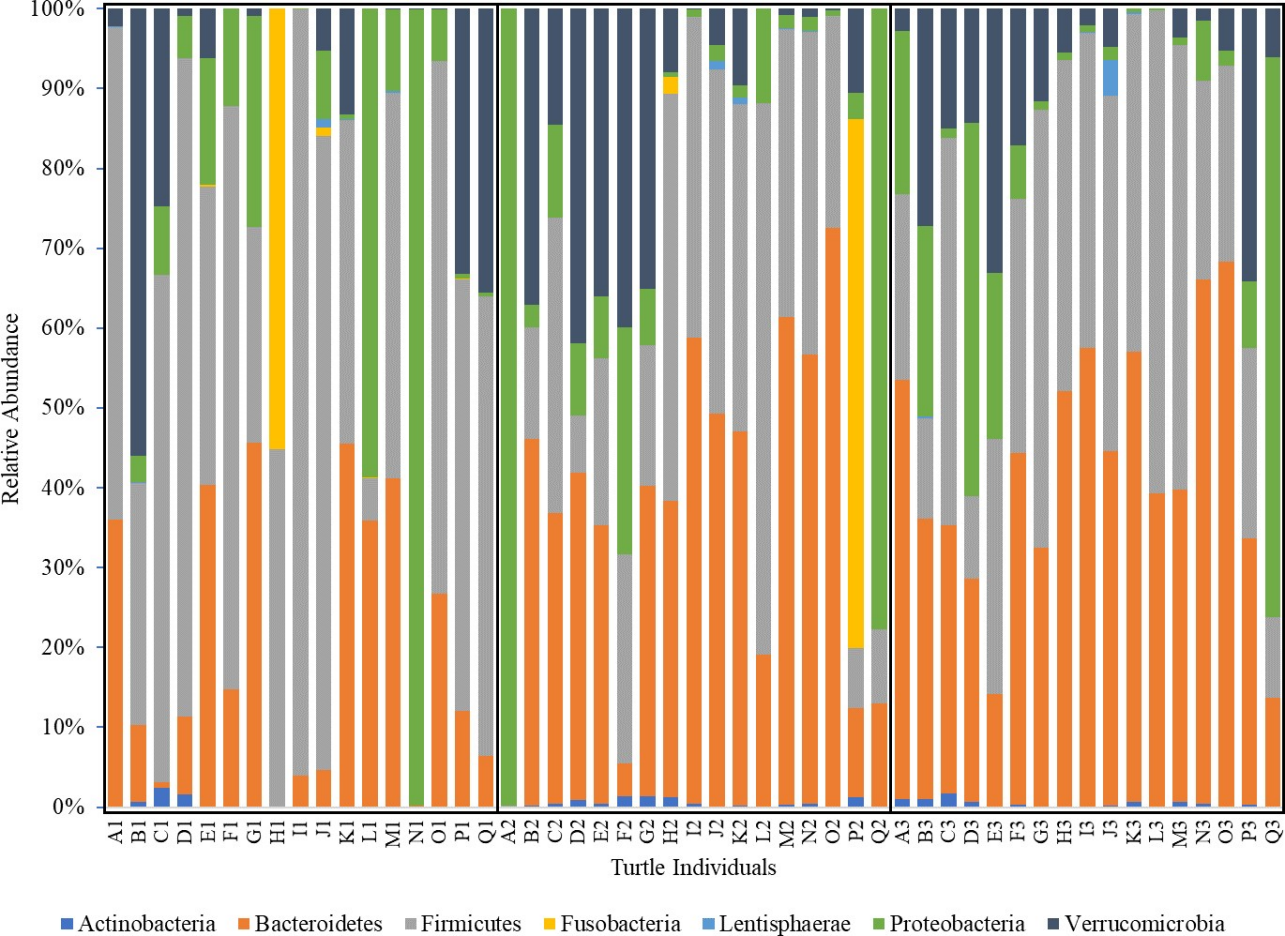
Relative abundance of bacterial phyla found in feces from juvenile green turtles across three time points in rehabilitation. Samples were collected at three time points throughout rehabilitation (admission, mid-rehabilitation, recovery).

The identified OTUs were assigned taxonomically and results shown are those present in at least 50% of sampled individuals (S1 Table). The dominant phyla changed across time points. At admission, the predominant phylum was Firmicutes (55.0%), followed by Bacteroidetes (11.4%) and Proteobacteria (6.2%), but by recovery, the Bacteroidetes (38.4%) and Firmicutes (31.8%) switched positions, and Verrucomicrobia (5.4%) outnumbered Proteobacteria (1.8%) (Fig 4).

At the family level, admission samples were predominately Lachnospiraceae (19.6%), Bacteroidaceae (10.7%), an unclassified family from the Clostridiales order (7.5%), and Ruminococcaceae (7.1%). Clostridiaceae and the unclassified family from Clostridiales were both significantly more abundant at admission than at other time points (both p = 0.041). Mid-rehabilitation samples were dominated by Bacteroidaceae (27.1%) followed by Lachnospiraceae (6.3%) and Porphyromonadaceae (5.6%). Recovery samples were primarily Bacteroidaceae (30.2%), Lachnospiraceae (18.5%), and Porphyromonadaceae (7.6%). Porphyromonadaceae were significantly more abundant at recovery than at other time points (p = 0.004; Table 2).

**Table 2:** Relative abundance of bacterial taxa in feces of juvenile green turtles across three time points in rehabilitation^a^.

	Median %			
Bacterial Taxa^b^	Admission	Mid-Rehab	Recovery	p-value^c^
**Family**				
Bacteroidaceae	10.7	27.1	30.2	0.357
Porphyromonadaceae	0.1	5.6	7.6	**0.004**
Unclassified family in Clostridiales	7.5	2.8	2.8	**0.041**
Clostridiaceae	4.9	0.4	0.7	**0.041**
Lachnospiraceae	19.6	6.3	18.5	0.319
Ruminococcaceae	7.1	2.9	4.8	0.193
Verrucomicrobiaceae	0.9	8	5.4	0.652
**Genus**				
*Bacteroides*	10.7	27.1	30.2	0.301
*Parabacteroides*	0.1	5.6	7.6	**0.005**
Unclassified genus in Clostridiales	7.5	2.8	2.8	**0.046**
Unclassified genus in Lachnospiraceae	12	4.6	17.4	0.301
Unclassified genus in Ruminococcaceae	6.1	2.2	4	0.109
*Akkermansia*	0.9	8	5.4	0.599

^a^Bacterial taxa shown were present in at least 50% of individual juvenile green turtles.

^b^Only bacterial taxa with a significant relative abundance (>3%) in turtles from at least one time point are represented.

^c^p-values have been adjusted for the false discovery rate. Bolded p-values were considered significant (p < 0.05).

The most common genera in admission samples were an unclassified genus in the Lachnospiraceae family (12%), followed by *Bacteroides* (10.7%) and an unclassified genus from the Clostridiales order (7.5%). The unclassified genus from the Clostridiales was significantly more abundant at admission than at other time points (p = 0.046). At mid-rehabilitation, the most common genera were *Bacteroides* (27.1%), *Akkermansia* (8%), and *Parabacteroides* (5.6%). By recovery, the most common genera were *Bacteroides* (30.2%), an unclassified genus in the Lachnospiraceae family (17.4%), and *Parabacteroides* (7.6%). *Parabacteroides* were significantly more abundant at recovery than at other time points (p = 0.005; Table 2).

#### Analysis of time point-specific bacterial communities

**Fig 5:**
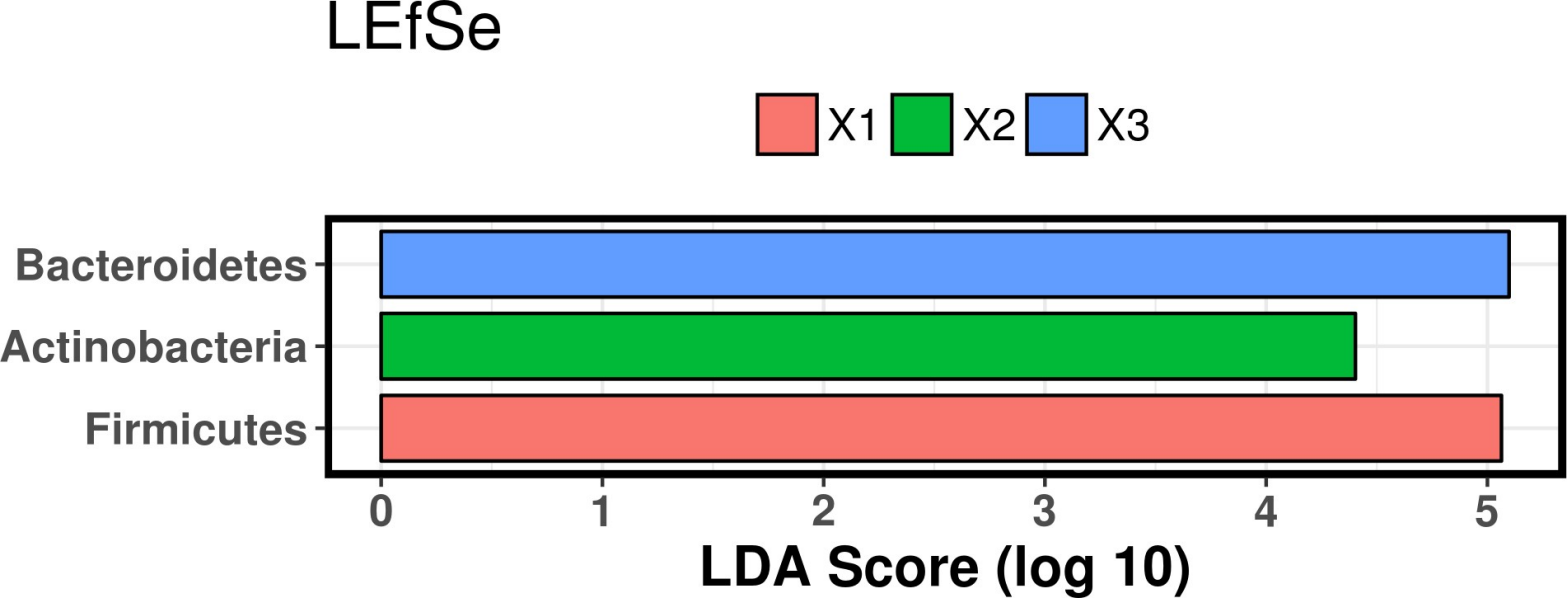
Linear discriminant analysis effect size (LEfSe) of bacterial phyla in feces from juvenile green turtles. Samples were collected at three time points throughout rehabilitation (X1 = admission, X2 = mid-rehabilitation, X3 = recovery).

**Fig 6:**
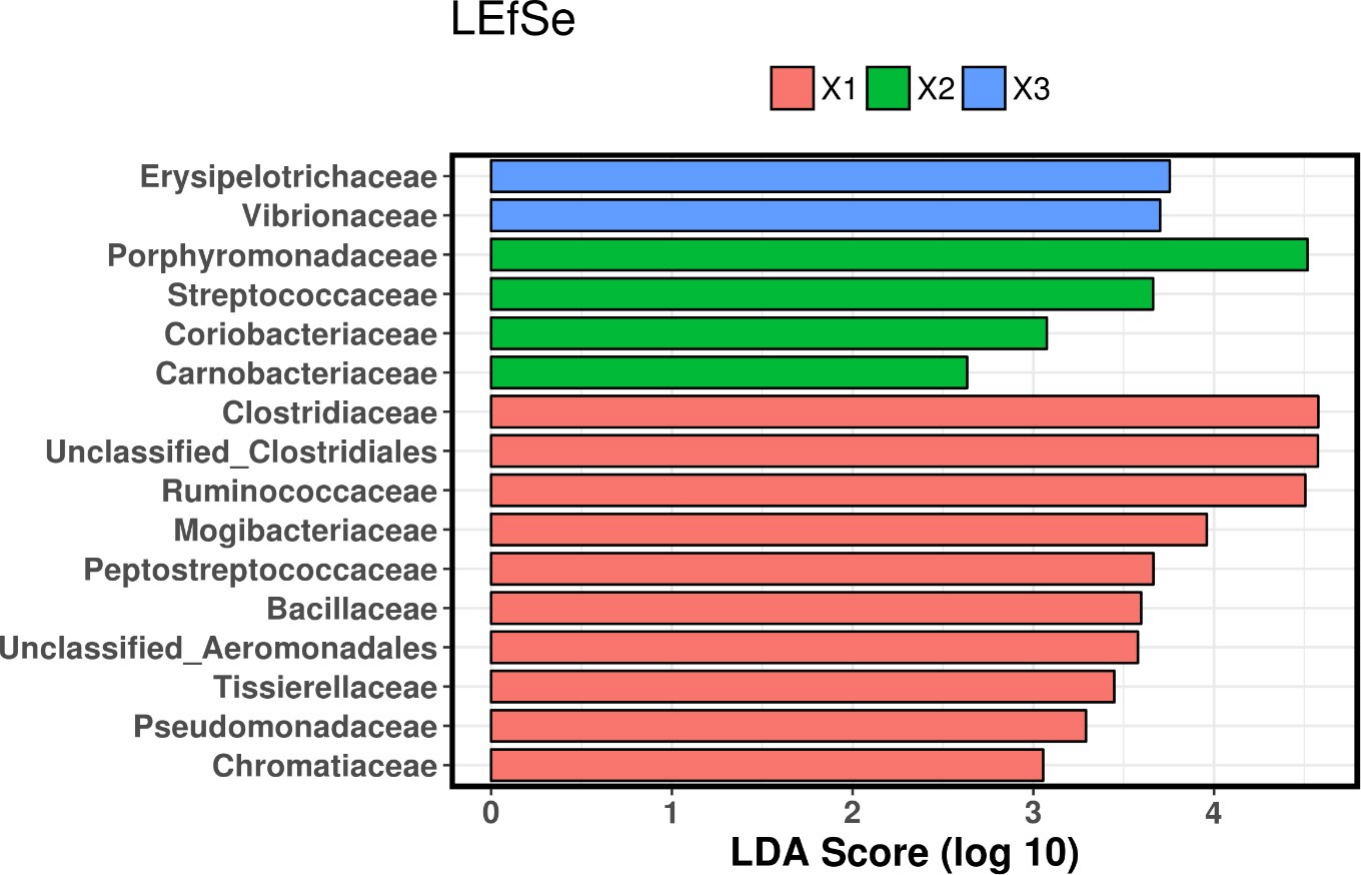
Linear discriminant analysis effect size (LEfSe) of bacterial families in feces from juvenile green turtles. Samples were collected at three time points throughout rehabilitation (X1 = admission, X2 = mid-rehabilitation, X3 = recovery).

LEfSe analysis revealed that the three time points were associated with significantly different bacterial taxa. At the phylum level, Firmicutes were most associated with turtles at admission, Actinobacteria at mid-rehabilitation, and Bacteroidetes at recovery (Fig 5). Bacterial families associated with admission turtles were Bacillaceae, Chromatiaceae, Clostridiaceae, Mogibacteriaceae, Peptostreptococcaceae, Pseudomonadaceae, Ruminococcaceae, Tissierellaceae, and unclassified families in the Clostridiales and Aeromonadales orders. The families Carnobacteriaceae, Coriobacteriaceae, Porphyromonadaceae, and Streptococcaceae were significantly associated with turtles at mid-rehabilitation, while at recovery, the families Erysipelotrichaceae and Vibrionaceae were most associated (Fig 6).

### Analysis of antibiotic administration effects

**Fig 7:**
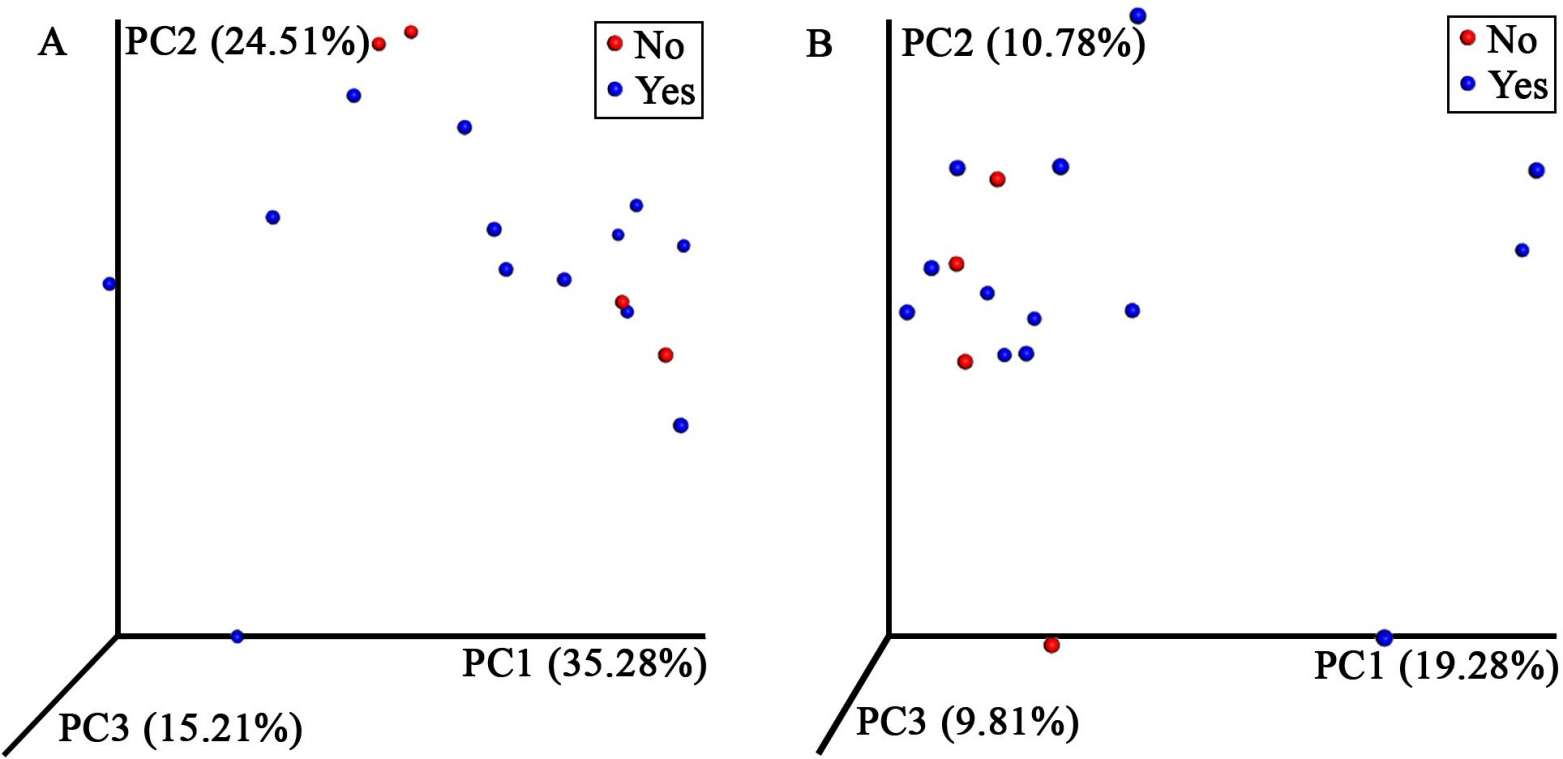
Juvenile green turtle fecal bacterial community composition in individuals on and not on antibiotics. Principle coordinate analysis of weighted (A) and unweighted (B) UniFrac distances of 16S rRNA genes found in feces from juvenile green turtles (*Chelonia mydas*) at admission, demonstrating that there was no significant difference in bacterial community composition between turtles that were (blue, N = 13) and were not (red, N = 4) on antibiotics (A: R = -0.087, p = 0.660; B: R = -0.193, p = 0.890). Each circle represents an individual fecal sample.

Because antibiotic use has been shown to influence the GI microbiota in humans [5], and sea turtles often receive antibiotics through the course of their rehabilitation, we explored whether antibiotic administration affected their GI microbiota. A range of antibiotic types were administered to different turtles based on individual needs, including: amikacin, ampicillin, ceftazidime, amoxicillin with clavulanic acid, enrofloxacin, and metronidazole (S2 Table). Thirteen of the 17 turtles received antibiotics by the time their admission sample was collected. There were no significant differences in observed OTUs, Shannon diversity index, or Chao 1 diversity indices among turtles at admission that did and did not receive antibiotics (Table 3; p = 0.865, p = 0.254, and p = 0.955, respectively). Principle coordinate analysis also failed to demonstrate a significant difference in bacterial community composition between turtles with or without antibiotic use (Fig 7).

**Table 3:** Juvenile green turtle fecal bacterial alpha diversity in individuals at the admission time point that did and did not receive antibiotics.

	Antibiotic use^a^		
	Yes (N = 13)	No (N = 4)	P-value^b^
**Observed OTUs**	250 (65–323)	232 (192–289)	0.865
**Shannon**	4.4 (2–5)	4.5 (3.9–5.2)	0.254
**Chao1**	362 (134–491)	349 (279–422)	0.955

^a^Values are presented as mean (range).

^b^Mann-Whitney statistical analysis

## Discussion

This is the first study to demonstrate a relationship between the green turtle fecal microbiome and different diets offered during the recovery process in a rehabilitation hospital. Fecal samples were assumed to be representative of the GI microbiome. This is a common assumption in the literature, although variation in community composition has been demonstrated among gastrointestinal sampling sites in horses, another hindgut fermenter [30]. In sea turtles, the most common sample types are feces and cloacal swabs; thus our results are comparable to other studies [10–13]. Our results show that regardless of diet, the fecal bacterial phyla of green turtles consists primarily of Firmicutes and Bacteroidetes, a feature that appears to be conserved across many vertebrate taxa, including other marine herbivores and green turtles elsewhere [11, 13, 31–34]. Principle coordinate analysis, however, revealed a clear difference in the community composition at admission compared to mid-rehabilitation and recovery, following the change in diet from primarily seafood to primarily vegetables. Most notably, relative abundances of the major phyla changed significantly–the microbiome of turtles at admission was primarily composed of Firmicutes with less Bacteroidetes, while the recovery time point reflected a significant decrease in Firmicutes, and an increase in Bacteroidetes. Other potential contributions to shifts in GI microbial composition over time in rehabilitation include the individual turtle’s health status at admission, time spent in rehabilitation, and stress associated with captivity. Future studies that control for health conditions and more strictly regulate the time on each diet would be helpful to identify shifts in GI microbiota that are affected by diet alone and are not confounded by other medical history and physical exam findings. This project involved rehabilitating animals protected under the Endangered Species Act, and thus these limitations could not be avoided.

While there was no significant difference in alpha diversity indices among the three time points, linear regression showed a significant decrease in Shannon diversity of the admission sample with increasing days in rehabilitation. That is, turtles that were in rehabilitation longer before the admission sample was taken had less diversity than turtles that were in rehabilitation for less time. This was not the case at the recovery time point, by which time alpha diversity was not significantly different than at admission. It is possible that diversity initially decreased in turtles due to the diet consumed early in rehabilitation (i.e. primarily seafood), and that diversity stabilized by the recovery time point, albeit with a different composition. No significant difference in alpha diversity yet significantly different community composition of the microbiome was also found in green turtles in Australia pre- and post-hospitalization, however that study did not look at the effect of length of time in rehabilitation or diets fed during that period [12].

The relative abundance of Firmicutes at admission is similar to what has been found in other free-ranging hindgut fermenters, and likely reflects the important role of this phyla in metabolizing seagrass and algae consumed by free-ranging green turtles [32–34]. Herbivorous marine iguanas (*Amblyrhynchus cristatus*) of the Galapagos Islands that have adapted to feed on macrophytic algae also have a microbiome composed predominately of Firmicutes (75.1%) and Bacteroidetes (8.2%) [33]. Much like green turtles, dugongs (*Dugong dugon*) are specialist marine herbivores that feed on seagrass, and their fecal bacteria phyla are also primarily Firmicutes (75.6%) and Bacteroidetes (19.9%) [34]. Florida manatees (*Trichechus manatus latirostris*), another hindgut fermenter but more of a generalist herbivore, also have a microbiome dominated by Firmicutes (77.3%) and Bacteroidetes (19.5%) [32].

Ahasan et al. (2017) found that the cloacal microbiome of healthy, wild-captured green turtles in Australia was primarily Firmicutes (>60%) and Bacteroidetes (>27%), while the dominant phyla in stranded turtles were Proteobacteria (47.6%), followed by Bacteroidetes (19.0%), Firmicutes (18.7%) and Fusobacteria (13.6%) [11]. Proteobacteria was also the dominant phylum in green turtles with presumed immunosuppression due to anthropogenic impacts in another study [13]. Both studies suggested that an abundance of Proteobacteria may be associated with poor health in green turtles. This was not the case in our study, where the relative abundance of Proteobacteria was relatively low (6.2%) in turtles admitted to rehabilitation, most of which were sick or physically traumatized, though the abundance did decrease over time in rehabilitation. Price et al. (2017) suggested that relative abundance of Proteobacteria may reflect the cloacal microbiome, which may be more influenced by the surrounding environment than the gut [14]. Campos et al. (2018), however, found significantly higher relative abundance of Proteobacteria in feces from captive green turtles fed a mixed macroalgae/fish diet compared to feces from wild green turtles [13]. This is similar to our study, in which turtles at the mid-rehabilitation time point consuming more animal protein had a higher relative abundance of Proteobacteria than turtles at recovery consuming 50% vegetables.

The only other study using feces in sea turtles was with loggerheads in rehabilitation (N = 3), and the bacterial community composition was Firmicutes (66%), Proteobacteria (23%) and Bacteroidetes (6.2%) [10]. The significance of the relatively high abundance of Firmicutes in a carnivorous species is unknown. However, in addition to diet, phylogeny plays a role in bacterial community composition, and closely related species generally have similar microbial communities [4]. This may explain why green turtles have a similar GI microbiome to loggerheads.

Regardless of time point, the Firmicutes phylum was almost entirely made up of Clostridia, which was also found in wild green turtles, loggerheads, and gopher tortoises [10, 11, 14, 35]. Similar to wild green turtles and gopher tortoises, Clostridia in our study included families known for their ability to metabolize cellulose (i.e. Ruminococcaceae, Clostridiaceae, and Lachnospiraceae) [11, 14, 35]. Each of these families was more abundant at admission than recovery, further supporting our conclusion that the microbiome at admission was reflective of wild turtles eating an herbivorous diet.

Mid-rehabilitation turtles in our study had a decreased proportion of Lachnospiraceae compared to admission and recovery turtles. This is likely due to the seafood offered early in rehabilitation, as Ahasan et al. (2018) found decreased abundance of Lachnospiraceae in green turtles fed strictly seafood in rehabilitation [12]. Other studies have shown that the relative abundance of Lachnospiraceae was greater than Ruminococcaceae in marine iguanas and green turtles eating macroalgaes, while the opposite was true in terrestrial herbivorous reptiles eating vascular plants [13, 33]. Campos et al. (2018) suggested that this could be related to differences in the cell wall structure of macroalgaes and vascular plants [13]. Green turtles in rehabilitation in the current study, however, were fed terrestrial vascular plants (i.e. lettuce, cucumbers, and green peppers), and these individuals also had higher relative abundance of Lachnospiraceae compared to Ruminococcaceae. In addition, Price et al. (2017), demonstrated that Lachnospiraceae were more abundant in green turtles in bay habitats consuming a predominately seagrass diet (a vascular plant), while Ruminococcaceae were more abundant in green turtles in pelagic habitats consuming animal/sargassum (a macroalgae) diets [14].

Campos et al. (2018) found no significant difference in the relative abundance of Ruminococcaceae and Lachnospiraceae in feces from wild green turtles compared to captive green turtles fed a mixed macroalgae/fish diet [13]. The authors suggested that this indicates that omnivory is unlikely to reduce the capacity of green turtles to digest plant material [13]. However, that study did not indicate the percentage of the captive diet that was macroalgae versus fish and did not indicate how long individuals had been in captivity. Thus, it is difficult to interpret these results compared to the current study, which found a significant difference in the microbiome of green turtles as they transitioned from a predominantly seafood to a 50% vegetable-based diet.

While turtles at recovery had significantly less Firmicutes, the class Erysipelotrichi within this phylum was significantly more abundant. Erysipelotrichi also increased in mice that were switched from a low-fat, plant-based diet to a high-fat/high-sugar diet [36]. The class Erysipelotrichi was primarily made up of the family Erysipelotrichaceae, which was also increased in another study of green turtles fed strictly seafood in rehabilitation [12].

The decrease in abundance of Firmicutes and increase in Bacteroidetes at recovery is likely because of abundant seafood intake early in rehabilitation and the low metabolic rate of turtles (individuals consumed up to 25% vegetables/75% seafood until mid-rehabilitation, averaging 54 days; at recovery, individuals were still consuming up to 50% seafood). The Bacteroidetes phylum consists of many bile-tolerant organisms that aid in protein digestion, and, in humans that switched from a fiber-rich diet to an animal protein-based diet, there was a decrease in Firmicutes and an increase in Bacteroidetes in as little as four days [6]. A similar shift occurred in a study of green turtles fed only seafood during rehabilitation: the GI microbiota at arrival were represented by Proteobacteria (33.6%), Firmicutes (25.5%), Bacteroidetes (14.4%) and Fusobacteria (9.1%), while after rehabilitation, the most abundant phylum was still Proteobacteria (36.9%), but this was followed by Bacteroidetes (25.4%), Fusobacteria (16.1%) and Firmicutes (14.2%) [12].

Future research into the time it takes for the green turtle GI microbiota to respond to diet would aid clinicians in deciding how long before release an individual should be transitioned to an herbivorous diet. Our results provide evidence that this transition should occur as soon as possible in rehabilitation to restore the normal GI flora prior to release. Fecal transfaunation and probiotic supplements could be considered, however there is no research on the effect of these on the GI microbiome of sea turtles. One co-author (Norton) has performed fecal transfaunation in green turtles with presumed benefits (unpublished data). The role of probiotics is controversial. Benefits, including the ability to increase the abundance of Firmicutes, have been noted in other chelonians [37, 38], however a study in mice and humans found that probiotics delayed return to normal gut microbiome composition post-antibiotics [39]. As next generation sequencing capabilities become more readily available and affordable, it is foreseeable that there will come a time when the fecal microbiome may be a practical diagnostic test that can be used to tailor the diet for optimum microbial composition.

The current study did not have feces from wild turtles as a control. Future research on the fecal microbiome of healthy, free-ranging green turtles would aid interpretation of these results. The effect of feeding commercially-available vegetables (i.e. cucumbers, green peppers, and lettuce) compared to algae and seagrass could be also be investigated. While it would be ideal to feed a natural diet, it is not currently feasible to produce seagrass and algae in sufficient quantities.

## Conclusions

In conclusion, once wild green turtles recruit to coastal waters, they feed on a primarily herbivorous diet of seagrass and algae. Nevertheless, many rehabilitation facilities offer a seafood-based diet early in rehabilitation to combat poor appetite and emaciation, and some hospitals and aquaria offer seafood constantly throughout captivity. Our results indicate that the GI microbiome of green turtles in rehabilitation changed significantly after consuming a seafood-based diet, from a community suited to metabolizing plant polysaccharides (i.e. the natural flora) to one adapted to digesting animal protein. These findings are important for providing this endangered species the best care possible, particularly rehabilitating animals which will be released back to the wild. If the GI microbiome is “primed” to digest seafood and not plants, green turtles may not acquire nutrients from their natural diet as efficiently. Knowledge of the ways in which the microbiome responds to diet, as shown in this study, are critical, so that individuals can be released in the best health possible to give them the optimum probability of survival.

## Supporting information

S1 Table Bacterial taxa present in at least 50% of individual green turtles across three time points in rehabilitation (i.e. admission, mid-rehabilitation, and recovery). Q-values are p-values that have been adjusted for the false discovery rate.
(PDF)

S2 Table Details on antibiotics administered to green turtles in rehabilitation at the Georgia Sea Turtle Center. Details include dosage, route, and dates of administration
(PDF)

S1 Fig Predominant bacterial genera immediately after defecation and 4 hours later. Samples from one individual comparing composition immediately after defecation to composition after 4 hours in salt water from the tank.
(TIFF)
