# Supplementary material for: The effect of diet on the gastrointestinal microbiome of juvenile rehabilitating green turtles (Chelonia mydas)
Source: PLoS One. 2020 Jan 15;15(1):e0227060. doi: 10.1371/journal.pone.0227060 (PMC6961862; doi:10.1371/journal.pone.0227060)
Supplement: S1 Table — Q-values are p-values that have been adjusted for the false discovery rate. (PDF) [file pone.0227060.s001.pdf]

S1 Table. Bacterial taxa present in at least 50% of individual green turtles across three time points in rehabilitation (i.e. admission, mid-rehabilitation, and recovery). Q-values are p-values that have been adjusted for the false discovery rate.

	Median			Range				
**Phylum**	**Admission**	**Mid-Rehab**	**Recovery**	**Admission**	**Mid-Rehab**	**Recovery**	**P-value**	**Q-value**
Actinobacteria	0	0.3	0.3	0-2.4	0-1.5	0-2.2	0.047	0.131
Bacteroidetes	11.4	37.9	38.4	0.1-46.1	0-73.4	13.7-70	0.056	0.131
Firmicutes	55	25.8	31.8	0-95.9	0.2-69.4	10.4-61.3	0.035	0.131
Fusobacteria	0	0	0	0-54.8	0-64.9	0-0	0.108	0.19
Lentisphaerae	0	0	0	0-1.1	0-0.9	0-4.7	0.67	0.782
Proteobacteria	6.2	3.3	1.8	0-99.8	0.5-99.7	0.1-69.9	0.79	0.79
Verrucomicrobia	0.9	8	5.4	0-55.6	0-40.8	0-33.9	0.526	0.737
**Class**								
Coriobacteriia	0.3	0.5	0.5	0-2.4	0-1.5	0-2.2	0.05	0.001
Bacteroidia	19.6	37	41.7	0.1-46.1	0-73.4	13.7-70	0.029	0.001
Bacilli	0	1.2	0.2	0-0.3	0-8.9	0-1.8	0.006	0.019
Clostridia	51	26.6	32.8	0-95.9	0-65.6	7.5-61.1	0.009	0.021
Erysipelotrichi	0.2	0.9	1.2	0-0.9	0-4.7	0.1-3.2	0	0.001
Fusobacteriia	3.3	3.9	0	0-54.8	0-64.9	0-0	0.272	0.408
[Lentisphaeria]	0.1	0.1	0.3	0-1.1	0-0.9	0-4.7	0.733	0.8
Alphaproteobacteria	0.1	0	0	0-1.9	0-0.1	0-0	0.08	0.159
Betaproteobacteria	0	0	0.1	0-0.7	0-0.3	0-1.1	0.655	0.786
Deltaproteobacteria	1.7	0.5	0.6	0-14.4	0-2.1	0-3	0.985	0.985
Gammaproteobacteria	13.1	15.2	11.8	0-99	0.2-99.4	0-69.7	0.118	0.202
Verrucomicrobiae	10.3	14	10.8	0-55.6	0-40.8	0-33.9	0.543	0.725
**Order**								
Coriobacteriales	0.3	0.5	0.5	0-2.4	0-1.5	0-2.2	0.05	0.099
Bacteroidales	19.6	37	41.7	0.1-46.1	0-73.4	13.7-70	0.029	0.086
Lactobacillales	0	1.1	0.2	0-0.1	0-8.9	0-1.6	0	0.002
Clostridiales	51	26.6	32.8	0-95.9	0-65.6	7.5-61.1	0.009	0.036
Erysipelotrichales	0.2	0.9	1.2	0-0.9	0-4.7	0.1-3.2	0	0.002
Fusobacteriales	3.3	3.9	0	0-54.8	0-64.9	0-0	0.272	0.408
Victivallales	0.1	0.1	0.3	0-1.1	0-0.9	0-4.7	0.733	0.8
Desulfovibrionales	1.4	0.5	0.6	0-14.4	0-2.1	0-3	0.985	0.985
Alteromonadales	1.2	0	0	0-7.9	0-0.4	0-0.2	0.491	0.652

Enterobacteriales	10.4	14.8	10.2	0-90.2	0-98.9	0-69.6	0.049	0.099
Vibrionales	0.7	0.3	1.4	0-2.6	0-3.8	0-18.3	0.074	0.127
Verrucomicrobiales	10.3	14	10.8	0-55.6	0-40.8	0-33.9	0.543	0.652
**Family**								
Coriobacteriaceae	0	0.3	0.3	0-2.4	0-1.5	0-2.2	0.05	0.108
Bacteroidaceae	10.7	27.1	30.2	0.1-46	0-71.5	7.1-63.9	0.238	0.357
Porphyromonadaceae	0.1	5.6	7.6	0-19.7	0-21.5	0-17.9	0.001	0.004
Rikenellaceae	0.1	0.4	0.2	0-2.1	0-3.6	0-2.8	0.387	0.489
Enterococcaceae	0	0	0	0-0	0-1.3	0-0.2	0.043	0.108
Streptococcaceae	0	0	0	0-0.1	0-8.9	0-1.6	0.012	0.041
Clostridiales;f__	7.5	2.8	2.8	0-41.4	0-8.4	0.6-10	0.012	0.041
Christensenellaceae	0	0	0	0-0.5	0-0	0-0.1	0.272	0.363
Clostridiaceae	4.9	0.4	0.7	0-38.5	0-61.7	0-6.7	0.01	0.041
Eubacteriaceae	0	0.1	0.1	0-3.2	0-1.6	0-1	0.983	0.985
Lachnospiraceae	19.6	6.3	18.5	0-51.1	0-39.5	3-47.3	0.199	0.319
Peptostreptococcaceae	1.1	0.2	0.4	0-4.2	0-0.9	0-2	0.036	0.108
Ruminococcaceae	7.1	2.9	4.8	0-37.7	0-10.2	1.1-14.3	0.105	0.193
Veillonellaceae	0	0	0	0-8.9	0-4.4	0-3.5	0.68	0.778
[Mogibacteriaceae]	0.8	0	0	0-14.7	0-0.3	0-0.3	0.001	0.004
[Tissierellaceae]	0	0	0	0-1.5	0-0	0-0	0	0.001
Erysipelotrichaceae	0	0.5	0.7	0-0.9	0-4.7	0.1-3.2	0	0.004
Fusobacteriaceae	0	0	0	0-54.8	0-64.9	0-0	0.272	0.363
Victivallaceae	0	0	0	0-1.1	0-0.9	0-4.7	0.733	0.8
Desulfovibrionaceae	0.1	0.3	0.4	0-14.4	0-2.1	0-3	0.985	0.985
Enterobacteriaceae	0.1	2.8	0.7	0-90.2	0-98.9	0-69.6	0.049	0.108
Pseudoalteromonadaceae	0.1	0	0	0-1.1	0-1.3	0-0.7	0.118	0.203
Vibrionaceae	0.1	0	0	0-2.6	0-2.5	0-18.2	0.087	0.175
Verrucomicrobiaceae	0.9	8	5.4	0-55.6	0-40.8	0-33.9	0.543	0.652
**Genus**								
Eggerthella	0	0.3	0.2	0-2.4	0-1.5	0-2.2	0.049	0.095
Bacteroides	10.7	27.1	30.2	0.1-46	0-71.4	7.1-63.9	0.238	0.301
Parabacteroides	0.1	5.6	7.6	0-19.7	0-21.5	0-17.9	0.001	0.005
Rikenellaceae;g__	0.1	0.4	0.2	0-1.8	0-3.6	0-2.6	0.232	0.301
Alistipes	0	0	0	0-0.5	0-0.1	0-0.4	0.301	0.37
Lactococcus	0	0	0	0-0.1	0-8.9	0-1.6	0.018	0.064

Clostridiales;f_;g	7.5	2.8	2.8	0-41.4	0-8.4	0.6-10	0.012	0.046
Christensenellaceae;g__	0	0	0	0-0.5	0-0	0-0.1	0.14	0.208
Clostridiaceae;g__	0.6	0.1	0.1	0-4.2	0-8.4	0-0.9	0.023	0.069
Clostridium	2.7	0.1	0.2	0-31.1	0-51.8	0-5.8	0.005	0.025
SMB53	0.3	0	0	0-25.3	0-1	0-0.8	0.005	0.025
Pseudoramibacter	0	0.1	0.1	0-3.2	0-1.6	0-1	0.7	0.734
Lachnospiraceae;g__	12	4.6	17.4	0-20.9	0-37.6	2.5-43.1	0.223	0.301
Anaerostipes	0	0	0	0-5	0-0.2	0-0.4	0.041	0.086
Blautia	0	0.2	0.1	0-0.6	0-1.2	0-1.3	0.001	0.005
Coprococcus	2.3	0.1	0.1	0-48.1	0-1.1	0-1.4	0.022	0.069
Dorea	0.1	0.3	0.3	0-7.1	0-3	0.1-1.6	0.125	0.199
Epulopiscium	0.3	0	0	0-6	0-7.8	0-1.4	0.076	0.137
Robinsoniella	0.2	0.1	0.1	0-2	0-1.7	0-1.1	0.856	0.856
Roseburia	0	0	0.1	0-1.6	0-1.3	0-3.5	0.559	0.601
[Ruminococcus]	0	0	0	0-1.3	0-0.2	0-0.1	0.717	0.734
Peptostreptococcaceae;g__	0.1	0.1	0.1	0-3.8	0-0.7	0-1	0.481	0.559
Clostridium	0.6	0	0	0-2.4	0-0.3	0-1.9	0.033	0.083
Ruminococcaceae;g__	6.1	2.2	4	0-21.8	0-8.5	1-13.6	0.058	0.109
Oscillospira	0.5	0.4	0.5	0-1.6	0-3.6	0.1-1.8	0.138	0.208
Ruminococcus	0.2	0	0	0-15.3	0-4.3	0-0.8	0.042	0.086
[Mogibacteriaceae];g__	0.8	0	0	0-14.7	0-0.2	0-0.3	0	0.005
Anaerovorax	0	0	0	0-0.3	0-0	0-0	0.006	0.027
Sedimentibacter	0	0	0	0-1.5	0-0	0-0	0	0.002
Erysipelotrichaceae;g__	0	0.2	0.2	0-0.7	0-1.7	0-3	0.001	0.005
Coprobacillus	0	0.1	0	0-0.2	0-1.2	0-1.4	0.195	0.28
[Eubacterium]	0	0	0	0-0.7	0-2.9	0-1.3	0.003	0.019
Bilophila	0	0.3	0.4	0-14.4	0-2.1	0-3	0.477	0.559
Enterobacteriaceae;g__	0	1.5	0.6	0-45.2	0-57.6	0-56.4	0.024	0.069
Citrobacter	0	0	0	0-1.4	0-4.5	0-1.9	0.026	0.07
Enterobacter	0	0	0.1	0-2.5	0-23.8	0-9.2	0.036	0.085
Hafnia	0	0	0	0-21.9	0-35.2	0-15.2	0.506	0.572
Morganella	0	0	0	0-57.6	0-11	0-2	0.237	0.301
Pseudoalteromonadaceae;g	0.1	0	0	0-1.1	0-1.3	0-0.7	0.041	0.086
Pseudoalteromonas	0	0	0	0-0.2	0-0.2	0-0.1	0.081	0.14
Photobacterium	0	0	0	0-0.2	0-0	0-0	0	0.005

Vibrio	0.1	0	0	0-1.7	0-2.5	0-18.2	0.105	0.174
Akkermansia	0.9	8	5.4	0-55.6	0-40.8	0-33.9	0.543	0.599
