# Supplementary material for: The effect of diet on the gastrointestinal microbiome of juvenile rehabilitating green turtles (Chelonia mydas)
Source: PLoS One. 2020 Jan 15;15(1):e0227060. doi: 10.1371/journal.pone.0227060 (PMC6961862; doi:10.1371/journal.pone.0227060)
Supplement: S2 Table — Details include dosage, route, and dates of administration (PDF) [file pone.0227060.s002.pdf]

S2 Table. Details on antibiotics administered to green turtles in rehabilitation at the Georgia Sea Turtle Center. Details include dosage, route, and dates of administration.

	Sample Date			
**Turtle ID**	**Admission**	**Mid-rehab**	**Recovery**	**Antibiotics Details**
A	1/10/2014	4/2/2014	5/6/2014	Ceftazidime 20 mg/kg SQ q3d 1/6/14-1/9/14, Amikacin 3 mg/kg SQ q3d 1/10/14-2/18/14 and 3/7/14-4/24/14, Clavamox 30 mg/kg PO SID 3/13/14-4/24/14
B	1/13/2014	2/26/2014	5/19/2014	Ceftazidime 20 mg/kg SQ q3d 1/9/14-4/24/14
C	1/15/2014	2/21/2014	3/9/2014	Ceftazidime 20 mg/kg SQ q3d 1/10/14-1/22/14
D	2/2/2014	2/23/2014	3/31/2014	Ceftazidime 20 mg/kg SQ q3d 1/30/14-3/28/14, Metronidazole 20 mg/kg PO SID 1/30/14-3/28/14
E	1/31/2014	2/28/2014	3/21/2014	Ceftazidime 20 mg/kg SQ q3d 2/1/14-3/12/14, Metronidazole 20 mg/kg PO SID 2/3/14-3/15/14
F	2/3/2014	2/26/2014	3/21/2014	Metronidazole 20 mg/kg PO SID 2/3/14-3/18/14, Ceftazidime 20 mg/kg SQ q3d 2/5/14-3/15/14
G	2/8/2014	3/4/2014	5/4/2014	Ceftazidime 20 mg/kg SQ q3d 2/8/14-4/6/14
H	4/3/2014	5/18/2014	8/21/2014	Amikacin 3 mg/kg SQ q3d 4/3/14-5/12/14 and 7/8/14-7/28/14
I	10/6/2014	11/10/2014	5/12/2015	None
J	10/8/2014	12/29/2014	5/27/2015	Ceftazidime 20 mg/kg SQ q3d 10/10/14-1/1/15
K	11/28/2014	3/14/2015	5/22/2015	Clavamox 30 mg/kg PO SID 3/10/15-4/2/15, Amikacin 3 mg/kg SQ q3d 3/13/15-4/2/15, Enrofloxacin 5 mg/kg PO SID 4/6/15-5/9/15
L	5/28/2015	6/15/2015	7/21/2015	Amikacin 3 mg/kg SQ q3d 3/21/15-5/4/15, Ceftazidime SQ 20 mg/kg q3d 5/7/15-6/8/15, Clavamox 30 mg/kg PO SID 5/8/15-6/8/15 and 6/28/15
M	5/3/2015	5/12/2015	6/4/2015	Amikacin 3 mg/kg SQ q3d 4/13/15-5/1/15
N	1/16/2016	2/20/2016	3/10/2016	Amikacin 3 mg/kg SQ q3d 12/13/15-1/30/16, Ampicillin 30 mg/kg SQ SID 12/23/15-12/26/15, Clavamox 30 mg/kg PO SID 12/27/15-2/1/16
O	12/12/2015	2/8/2016	3/10/2016	Oxytetracycline at NEAQ*, Amikacin 3 mg/kg SQ q3d 12/14/15-1/24/16
P	2/3/2016	3/1/2016	4/9/2016	Amikacin 3 mg/kg SQ q3d 1/24/16-3/7/16, Metronidazole 20 mg/kg PO SID 3/12/16-4/3/16
Q	1/31/2016	4/16/2016	4/28/2016	Amikacin 3 mg/kg SQ q3d 1/26/16-4/10/16, Clavamox 30 mg/kg PO SID 2/2/16-4/12/16

* NEAQ – New England Aquarium. This individual was transferred from the NEAQ and received oxytetracline while at NEAQ.
