# Supplementary figures and images for: The effect of diet on the gastrointestinal microbiome of juvenile rehabilitating green turtles (Chelonia mydas)
Source: PLoS One. 2020 Jan 15;15(1):e0227060. doi: 10.1371/journal.pone.0227060 (PMC6961862; doi:10.1371/journal.pone.0227060)

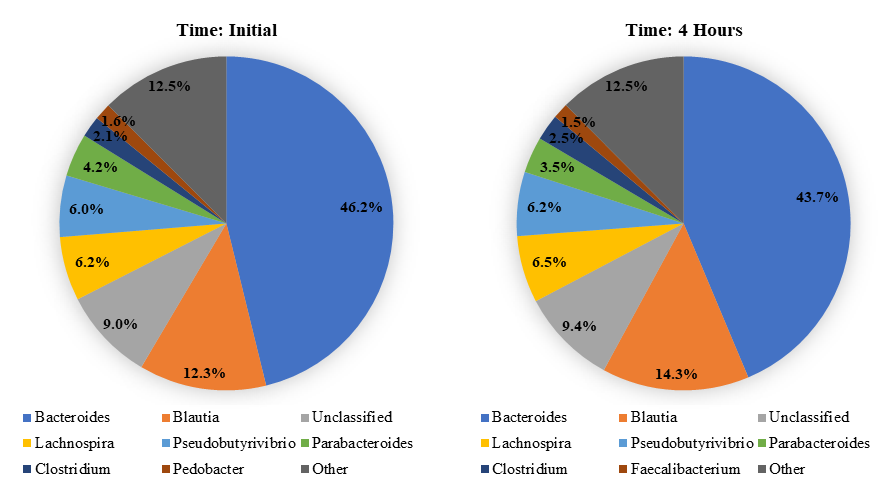

Supplement: S1 Fig — Samples from one individual comparing composition immediately after defecation to composition after 4 hours in salt water from the tank. (TIFF) [file pone.0227060.s003.tiff]
